# The Effectiveness of Mindfulness-Based Interventions for Anxiety Disorders in Adults: A Systematic Narrative Review

**DOI:** 10.1192/bjo.2022.197

**Published:** 2022-06-20

**Authors:** Lisa Harrison, Katja Umla-Runge, Athanasios Hassoulas

**Affiliations:** Cardiff University, Cardiff, United Kingdom

## Abstract

**Aims:**

In recent years there has been accelerated clinical interest in Mindfulness based interventions (MBI's) leading to an upswell in research due the impact of its wide clinical application. Mindfulness Based Cognitive Therapy (MBCT) and Acceptance and Commitment Therapy (ACT) have recently been investigated for the beneficial treatment of anxiety-based disorders in adults. The aim of the current review was to appraise and synthesise findings of studies published within the last decade, in determining the efficacy of MBCT and ACT in treating anxiety disorders in adults, given gaps identified in the existing literature.

**Methods:**

Scoping searches were conducted using MEDLINE, PsycINFO, Emcare, and Cochrane databases. The Synthesis Without Meta-analysis protocol (SWiM) was adopted for this review, in evaluating the efficacy of MBCT and ACT for anxiety disorders in adults. The review was conducted in accordance with the Preferred Reporting Items for Systematic Reviews and Meta-Analyses (PRISMA) Standards.

**Results:**

The results of this review suggest that MBCT and ACT are effective therapeutic modalities in improving anxiety in adult populations. The results are, however, tentative. Whilst both MBI's show promise in the treatment of anxiety disorders, with the paucity of existing systematic reviews and methodological flaws within overall primary study design, the results should be interpreted with caution.

**Conclusion:**

The overall therapeutic effectiveness of MBI's has been assessed and the general data support its efficacy. However, a judicious approach is required as results continue to remain inconclusive grounded in the totality of the evidence.

The current review revealed the ongoing methodological concerns encountered in determining the comparative effectiveness of MBCT and ACT for anxiety disorders in adults. Due to the current limited number of comparative studies of mindfulness *based* with mindfulness *informed* interventions, it could be suggested that a lack of systematic research is slowly influencing a collective understanding of MBI's being a homogenous group of treatments. The lack of delineation can have an impact on research, clinical practice and policy making. Further high quality research is required to continue to bridge the science practice gap. Without depth of understandings associated with the mechanisms of change and the impact that contextual aspects have on the outcome effectiveness, there are significant implications for practice and patient care. It is of importance that the adaptation and subsequent developments in clinical practice do not outpace the research base to fully understand the mechanisms that make each MBI effective, for which population and diagnoses.

